# Projected population exposure to heatwaves in Xinjiang Uygur autonomous region, China

**DOI:** 10.1038/s41598-024-54885-1

**Published:** 2024-02-25

**Authors:** Diwen Dong, Hui Tao, Zengxin Zhang

**Affiliations:** 1 College of Ecology and Environment, Xinjiang University, Urumqi, 830017 China; 2 State Key Laboratory of Desert and Oasis Ecology, Xinjiang Institute of Ecology and Geography, Chinese Academy of Sciences, Urumqi, 830011 China; 3 Joint Innovation Center for Modern Forestry Studies, College of Forestry, Nanjing Forestry University, Nanjing, 210037 China; 4 Institute of Statistics and Data Science, Xinjiang University of Finance & Economics, Urumqi, 830012 China; 5 University of Chinese Academy of Sciences, Beijing, 100049 China

**Keywords:** Climate sciences, Environmental sciences, Natural hazards

## Abstract

The intensification of heatwaves dues to climate change is a significant concern, with substantial impacts on ecosystems and human health, particularly in developing countries. This study utilizes NASA Earth Exchange Global Daily Downscaled Projections (NEX-GDDP-CMIP6) and projected population data accounting for China’s population policies to project changes in various grades of heatwaves (light, moderate, and severe) and the population exposure to heatwaves (PEH) in Xinjiang under three shared socioeconomic pathways (SSP1–2.6, SSP2-4.5, and SSP5-8.5). The results show that the number of days and intensity of heatwaves in Xinjiang are projected to increase. Heatwaves occurring in Xinjiang will predominantly be severe heatwaves (SHW) in the long-term under the SSP5-8.5 scenario, and the number of SHW days projected to increase by 62 ± 18.4 days compared to the reference period. Changes in heatwaves are anticipated to influence PEH, estimating population exposure to light, moderate, and severe heatwaves (LPEH, MPEH, and SPEH) at 534.6 ± 64 million, 496.2 ± 43.5 million, and 1602.4 ± 562.5 million person-days, respectively, in the long-term under the SSP5-8.5 scenario. The spatial distribution of PEH is projected to be consistent with that of the reference period, with high values persisting in Urumqi, Kashgar and Hotan. Changes in PEH are primarily driven by climate effects, followed by interactive effects, while population effects contribute the least. Therefore, mitigating climate change is crucial to reduce the PEH in Xinjiang.

## Introduction

Heatwaves are extreme weather events that have negative impacts on the human health and social economy^1,2^. For example, the European heatwave in the summer of 2003 caused in excess of 70,000 deaths^3^. In 2017, exceptional heatwaves in China killed over 16 thousand lives and resulted in economic losses estimated at approximately 61.3 billion RMB^4^. Numerous studies have shown that these catastrophic consequences would consistently increase with climate change^5^. The Intergovernmental Panel on Climate Change (IPCC) states that the risks associated with climate change are determined not only by the hazards but also by the exposure to those hazards^6^. Therefore, projecting changes in heatwaves and population exposure to heatwaves (PEH) is critical to developing policies for heatwave mitigation and adaptation.

Population exposure is commonly considered a function of both climate and population^7^. Numerous studies have investigated the impacts of various extreme weather events, including droughts^8^, floods^9^, extreme precipitation^10^, and heatwave^11^ on human health using population exposure as the metric. These studies have consistently emphasized significant increase in population exposure to extreme events in the future. For instance, Chen et al.^12^ find significant increase in population exposure to extreme precipitation across global lands, with projections of increase by at least 50% in the future under the Shared Socioeconomic Pathway (SSP) 5–8.5 scenario. Additionally, Wang et al.^13^ indicate that global urban population exposure to heatwaves will escalate under four SSP scenarios (SSP1-2.6, SSP2-4.5, SSP3-7.0, and SSP5-8.5). Changes in population exposure depend on climate change and the size and distribution of population^14^. However, some previous studies have ignored population changes and relied on static population data for projecting changes in population exposure. For instance, Sun et al.^15^ use regional climate model, COSMO-CLM, with 2010 population data to assess population exposure to drought in the Haihe River Basin in a warming world. Lyon et al.^16^ quantify population exposure to contiguous heatwave regions over the US from 2031 to 2055, based on climate model projections and static population in 2015. This approach may potentially influence the accuracy of population exposure assessment since both climate change and population are critical factors influencing exposure^17^. Thus, taking population changes into account when projecting changes in PEH will produce more reliable estimates.

The threat of heatwaves to human health seems almost unavoidable^18^. As global warming continues, the threat is projected to increase^19^. Populations living in developing countries are more susceptible to the effect of heatwaves than those in developed countries due to population growth rates and economic levels^20,21^. As the most populous developing country in the world, China is particularly susceptible to climate extremes^22,23^. China has experienced severe heatwaves, resulting in substantial human and socioeconomic losses over recent decades^24^. Previous studies have indicated that heatwaves tend to have more severe impacts on economically advanced and densely populated regions^25^. Previous researches on heatwaves in China have predominantly focused on Eastern and Southern China because of the high population density and economic development of these regions^26,27^. Such as Zhang et al.^28^ quantify future changes in heatwaves in Eastern China and find that the frequency, duration, and magnitude of heatwaves would increase approximately 5, 4, and 20 times, respectively, compared with the period 1986–2005. Although the sparsely populated Northwest China is particularly sensitive to the effects of heatwaves, limited attention has been paid to the region. People in Northwest China may be more vulnerable than those in Eastern China and Southern China due to the backward economy and limited healthcare services^29^. Recent studies have suggest that global warming will lead to the increase in heatwaves across China, with the Northwest region experiencing the most significant rise^30^. Due to climate factors, the characteristics of heatwaves in Northwest China is significantly different from those in Eastern China and Southern China, rendering climate change policies suitable for the latter regions unsuitable for the former^31^. Therefore, the in-depth study of heatwaves in Northwest China is necessary to provide decision makers with accurate information on regional climate policies for adaptation and mitigation.

Xinjiang, located in Northwest China, serves as the core area for the ‘Belt and Road’^32^. As the important part of the Central Asia arid zone, Xinjiang’s fragile ecosystem and agriculture-based economy make it particularly sensitive to climate change^33^. Over the past few decades, frequent heatwaves have occurred in most regions of Xinjiang^34^. In the summer of 2015, Xinjiang suffered an unprecedented heatwave. This led to a reduction in crop yields and quality, and an increase in the number of patients suffering from airway diseases and cerebrovascular diseases^35^. According to studies investigating future changes of heatwaves in China, Xinjiang is projected to suffer more frequent and intense heatwaves in the twenty-first century^36^. Although the population density in Xinjiang is lower than that of most parts of China, its rapid population growth and concentrated distribution pattern are projected to more people will be affected by frequent heatwaves. In general, the combination of high temperatures and high relative humidity increases the perceived temperature on the human body^28^. However, most existing studies that project heatwaves have only considered air temperature^36,37^. Since the mid-1980s, Xinjiang's climate has undergone the shift from warm-dry to warm-humid, and neglecting relative humidity when identifying heatwaves in Xinjiang may lead to an underestimation of their intensity and impacts^38^. Therefore, for the comprehensive projection of variations in heatwaves and PEH in Xinjiang, it is crucial to consider the combination of air temperature and relative humidity.

This study focuses on the future changes of different grades of heatwaves and associated population exposure in Xinjiang under different scenarios. The specific objectives of this study are: (1) to evaluate the simulation capability of 16 climate models to reproduce the interannual variability and spatial pattern of heatwaves in Xinjiang during the reference period, (2) to project changes in the number of days and relevant population exposure for different grades of heatwaves under three SSP scenarios (SSP1-2.6, SSP2-4.5, and SSP5-8.5), and (3) to reveal the relative importance of climate, population and their interactions on changes in exposure. The results of this study can provide a scientific basis for the development of disaster prevention and mitigation policies in arid regions in response to future climate change.

## Data and methods

### Study area

**Figure 1:**
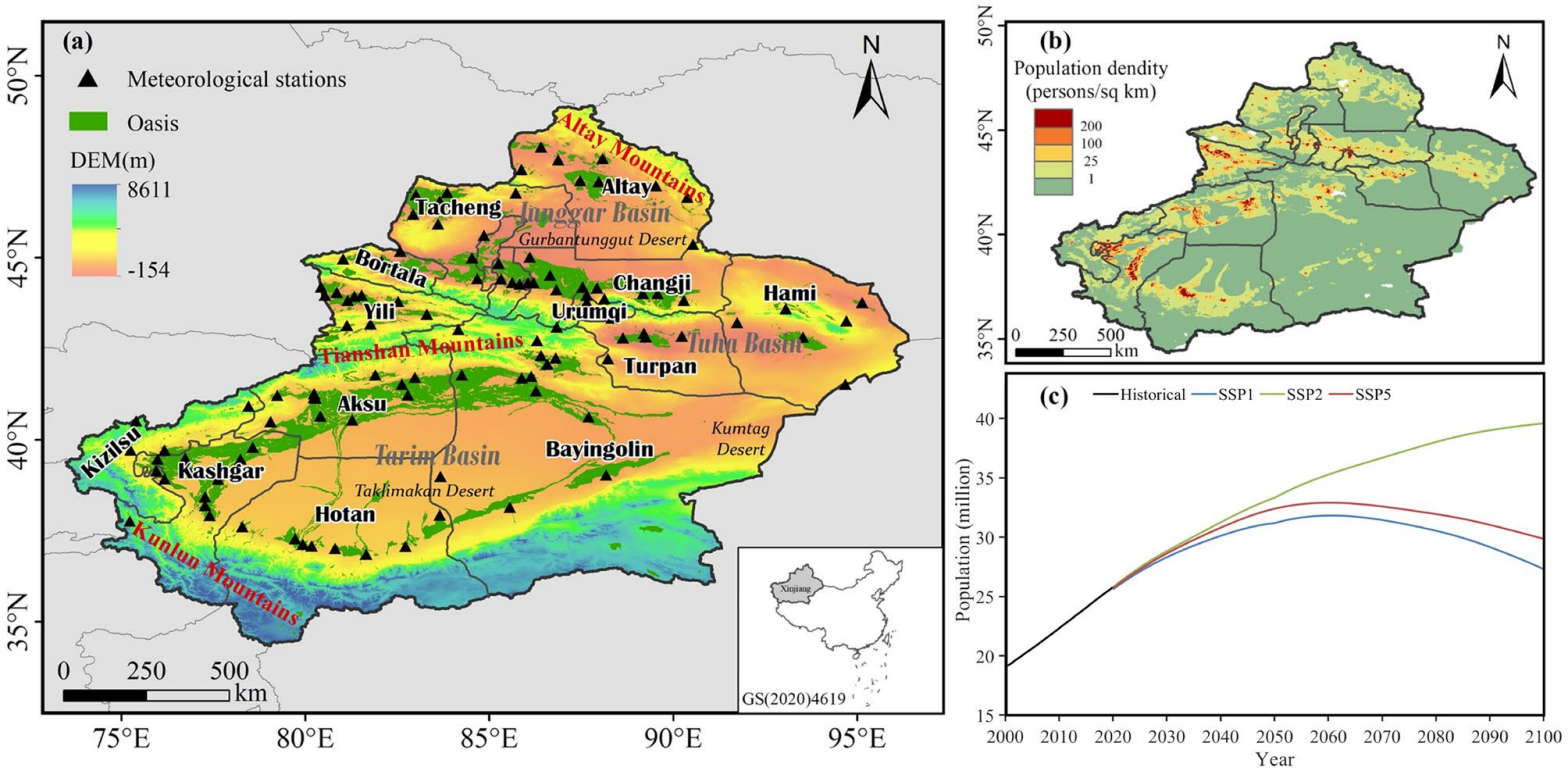
Overview of the study area. (**a**) Topography of Xinjiang. (**b**) Spatial distribution of population density in 2000. (**c**) Population in Xinjiang from 2000 to 2100. The maps in figure are created using ArcGIS v10.6 (https://www.esri.com/).

Xinjiang is the largest province in China, with a total area of 1.66 million km^2^ (Fig. 1). Situated in the hinterland of the Eurasian continent, it is an important part of the Central Asia arid zone. The region is far from the source of water vapor and has a typical temperate continental climate. The unique mountain-basin system in Xinjiang renders its climate complex and very sensitive to climate change^39^.

According to data from the seventh national census conducted in 2020, the population in Xinjiang is recorded at 25.85 million. Influenced by natural factors, the spatial distribution of population in Xinjiang is highly uneven, with typical characteristics of population distribution in arid zone^40^. More than 90% of the population is distributed in oases, with higher population density distribution in regions such as Urumqi, Yili and Kashgar.

### Dataset

The observational daily maximum temperature and relative humidity in this study are obtained from the gridded daily scale dataset of CN05.1, which are provided by National Climate Center, China Meteorological Administration. The dataset is constructed by anomaly approach method based on 2416 meteorological stations in China, with the spatial resolution of 0.25° × 0.25°. The dataset has been quality controlled and widely applied as the reference to evaluate and calibrate model simulations^41^.

The climate models dataset are derived from the latest version of the NASA Earth Exchange Global Daily Downscaled Projections dataset (NEX-GDDP-CMIP6, https://www.nccs.nasa.gov/services/data-collections/land-based-products/nex-gddp-cmip6). The dataset is based on output from the CMIP6, using downscaling and bias correction/spatial disaggregation method to obtain high-resolution daily gridded meteorological dataset with the resolution of 0.25° × 0.25°^42^. The dataset has been used extensively in regional studies of extreme weather events^43,44^. In this study, we use 16 climate models to project heatwaves in Xinjiang (Table 1). The multi-model ensemble (MME) approach can effectively reduce the uncertainty of the simulations^45^. Therefore, in order to improve the accuracy and reliability of projection, the MME of 16 climate models is used in this study.

**Table 1:** Details of the selected NEX-GDDP-CMIP6 models.

Id	Model name	Country	Id	Model name	Country
1	ACCESS-CM2	Australia	9	INM-CM5-0	Russia
2	ACCESS-ESM1-5	Australia	10	IPSL-CM6A-LR	France
3	CanESM5	Canada	11	MIROC6	Japan
4	CMCC-ESM2	Italy	12	MPI-ESM1-2-HR	Germany
5	EC-Earth3	Sweden	13	MPI-ESM1-2-LR	Germany
6	EC-Earth3-Veg-LR	Sweden	14	MRI-ESM2-0	Japan
7	GFDL-ESM4	USA	15	NorESM2-LM	Norway
8	INM-CM4-8	Russia	16	NorESM2-MM	Norway

The population data from 2021 to 2100 are taken from the Provincial and gridded population projection for China under shared socioeconomic pathways from 2010 to 2100 (10.6084/m9.figshare.c.4605713). The dataset is produced taking into account the population policies implemented in China in recent years (fertility promoting policies and population ceiling restrictions of megacities), and is the high-resolution gridded data (1 km × 1 km) that better matches the actual situation in China^46^. The population datasets are upscaled to the precision of 0.25° by summation method to match with the climate data.

## Methods

### Definition of heatwaves

Considering the impact of heatwaves on human health, the heatwave index (HI), which combines air temperature and relative humidity, is utilized in this study as an indicator for identifying heatwaves^47^. The HI can be calculated as follows:

1 $$HI=1.2\times \left(TI-{TI}{\prime}\right)+0.35\sum_{i=1}^{N-1}1/{nd}_{i}\left({TI}_{i}-{TI}{\prime}\right)+0.15\sum_{i=1}^{N-1}1/{nd}_{i}+1 .$$

In which $TI$ denotes the torridity index of the current day, ${TI}{\prime}$ is the critical value of torridity index, ${TI}_{i}$ represents the torridity index of the $i$-th day before the current day, ${nd}_{i}$ is the number of days from the $i$-th day to the current day, and $N$ is the duration of hot weather process (days).

The $TI$ can be obtained as follows:

2 $$TI=1.8\times {T}_{max}-0.55\times \left(1.8\times {T}_{max}-26\right)\times \left(1-0.6\right)+32 RH\le 60\%,$$

3 $$TI=1.8\times {T}_{max}-0.55\times \left(1.8\times {T}_{max}-26\right)\times \left(1-RH\right)+32 RH>60\% ,$$

where ${T}_{max}$ is the daily maximum temperature (℃), $RH$ represents the daily relative humidity (%).

The critical value of torridity (${TI}{\prime}$) is used to determine if the weather is hot. If $TI$ exceeds ${TI}{\prime}$, it indicates that the day is hot weather. ${TI}{\prime}$ is calculated using the quantile method with the following formulas:

4 $$\widehat{{Q}_{i}}\left(p\right)=\left(1-\gamma \right){X}_{\left(j\right)}+\gamma {X}_{\left(j+1\right)},$$

5 $$j=int\left(p\times n+\left(1+p\right)/3\right),$$

6 $$\gamma =p\times n+\left(1+p\right)/3-j ,$$

where $\widehat{{Q}_{i}}\left(p\right)$ represents the $i$-th quantile value, $p$ is the quantile (0.5 in this study), $n$ is the length of $TI$ series, $j$ is the $j$-th $TI$, $X$ denotes the sample sequence of the $TI$ in ascending order.

According to the magnitude of the HI, Heatwaves are graded into light, moderate, and severe heatwaves (LHW, MHW, and SHW). The classification standard is shown in Table 2.

**Table 2:** Classification of heatwaves.

Grades	Heatwave index
Light heatwave (LHW)	2.8 ≤ HI < 6.5
Moderate heatwave (MHW)	6.5 ≤ HI < 10.5
Severe heatwave (SHW)	HI ≥ 10.5

### Metrics of model performance

The interannual variability skill score (IVS)^48^ is used to evaluate the interannual variability of the simulations compared to the observations, which is expressed as:

7 $$IVS={\left(\frac{{STD}_{m}}{{STD}_{o}}-\frac{{STD}_{o}}{{STD}_{m}}\right)}^{2} ,$$

where ${STD}_{o}$ and ${STD}_{m}$ are the interannual standard deviations of the observation and simulations, respectively.

To evaluate the performance of the model in reproducing the spatial pattern of heatwaves, the Distance between Indices of Simulation and Observation (DISO)^49^ is used. For the observed values (A = (a_1_, a_2_,…, a_n_)) and the model-simulated values (B = (b_1_, b_2_,…, b_n_)), the DISO can be calculated as follows:

8 $$DISO=\sqrt{{\left(R-1\right)}^{2}+{\left(AE\right)}^{2}+{\left(RMSE\right)}^{2}},$$

9 $$R=\frac{\sum_{i=1}^{n}\left({a}_{i}-\overline{a }\right)\left({b}_{i}-\overline{b }\right)}{\sqrt{{\sum }_{i=1}^{n}{\left({a}_{i}-\overline{a }\right)}^{2}}\sqrt{{\sum }_{i=1}^{n}{\left({b}_{i}-\overline{b }\right)}^{2}}} ,$$

10 $$AE=\frac{1}{n}\sum_{i=1}^{n}\left({b}_{i}-{a}_{i}\right),$$

11 $$RMSE=\sqrt{\frac{1}{n}\sum_{i=1}^{n}{\left({b}_{i}-{a}_{i}\right)}^{2}},$$

where $\overline{a }$ and $\overline{b }$ is the mean of A and B, respectively. The values of IVS and DISO closer to 0 indicate that the model is better model performance.

### Weighting methodology

MME is used with no rules for determining the number of models to be used, and variations in model weighting schemes are used in different studies^50^. Based on the overall performance of the model simulations, to give the highest weight to the best performing models, this study uses performance weighting to generate MME. The weights then have the value:

12 $${W}_{i}=\frac{{R}_{i}}{\sum_{i=1}^{N}{R}_{i}},$$

13 $${R}_{i}=\frac{\sum_{i=1}^{N}{S}_{i}}{{S}_{i}},$$

where ${S}_{i}$ is the sum of the rankings of the model's simulated interannual variability and spatial patterns.

### Population exposure to heatwaves

Population exposure to heatwaves (PEH) is defined as the number of people exposed to heatwaves, is generally calculated by multiplying the population in each grid cell by the number of heatwave days^14^. Therefore, the unit of PEH is person-days. This study focuses on PEH for three periods including near-term (2021–2040), mid-term (2041–2060), and long-term (2081–2100) under three SSP scenarios (SSP1-2.6, SSP2-4.5, and SSP5-8.5).

According to the definition of PEH, changes in PEH are affected by climate effect, population effect and interactive effect. To evaluate the impact of these effects on future PEH changes, we calculate the relative contribution of each effect according to the approach of Jones et al.^14^. The relative contribution of each effect is calculated as follows:

14 $${CR}_{cli}=\frac{{P}_{r}\times \Delta H}{{H}_{r}\times \Delta P+{P}_{r}\times \Delta H+\Delta H\times \Delta P}\times 100\% ,$$

15 $${CR}_{pop}=\frac{{H}_{r}\times \Delta P}{{H}_{r}\times \Delta P+{P}_{r}\times \Delta H+\Delta H\times \Delta P}\times 100\% ,$$

16 $${CR}_{int}=\frac{\Delta H\times \Delta P}{{H}_{r}\times \Delta P+{P}_{r}\times \Delta H+\Delta H\times \Delta P}\times 100,$$

where ${P}_{r}$ is the population from the base-year of 2000 (person), ${H}_{r}$ indicate the annual days of heatwave (days) in the reference period (1995–2014), $\Delta H$ and $\Delta P$ are the change in the number of heatwave days and population in the future period compared to the reference period. ${CR}_{cli}$, ${CR}_{pop}$, and ${CR}_{int}$ represent the contribution rates of changes in the climate, population, and their interactions, respectively.

## Results

### Changes in historical heatwaves and population exposure

**Figure 2:**
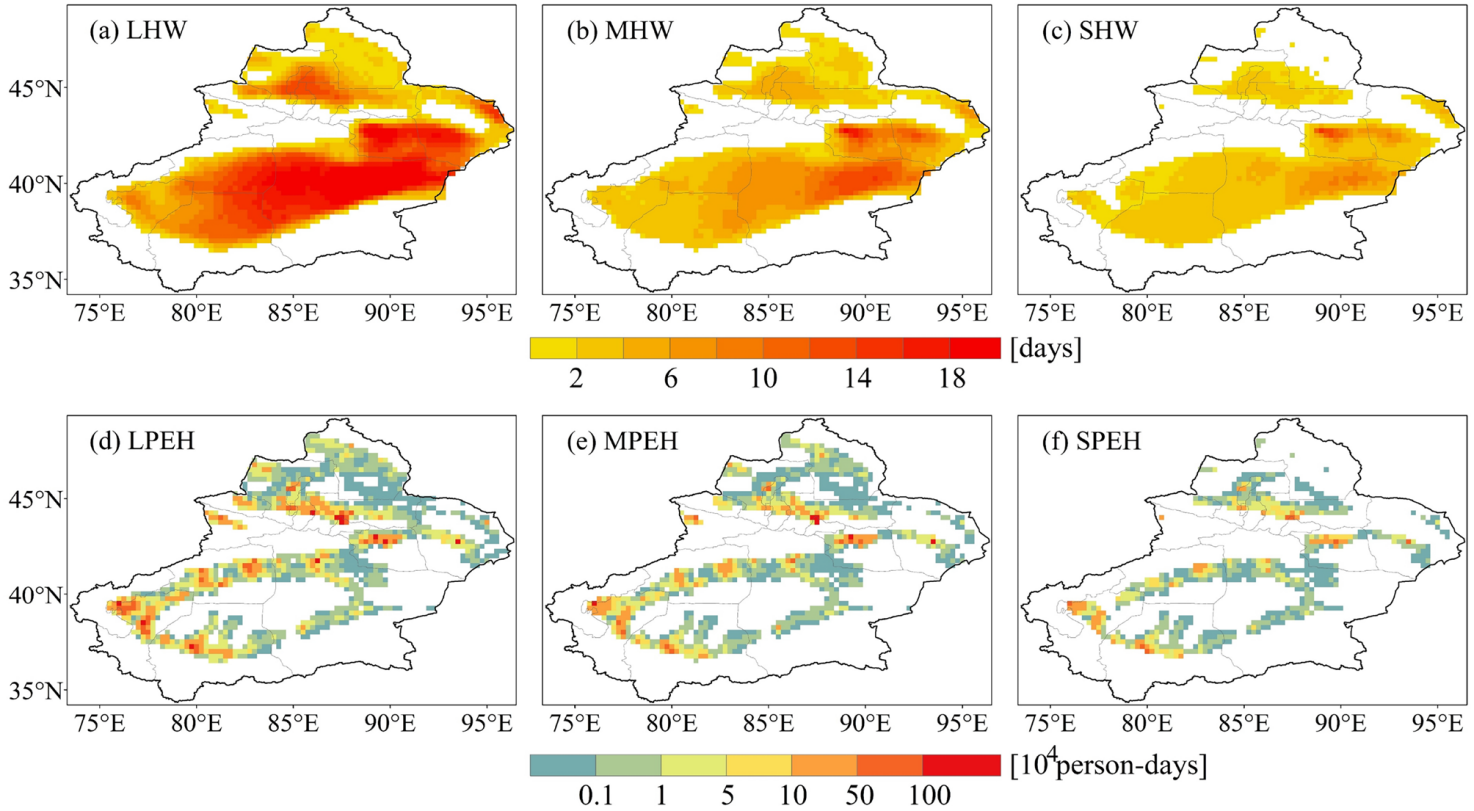
Spatial distribution of different grades of heatwave days and population exposure to heatwaves in Xinjiang during the historical period.

The spatial distribution of heatwave days for different grades during the reference period is presented in Fig. 2a–c. The observational results show that the spatial distribution of heatwaves is closely associated with the topographic features, mainly distributed in basin areas. LHW exhibit the broadest spatial extent and the greatest number of days (Figs. 2a). The number of LHW days ranges from about 0–23 days, with only some regions of Turpan and the Kumtag desert having more than 19 days. MHW affect smaller geographical area compared to LHW, with the number of heatwave days varying from 0 to 18 days (Figs. 2b). Regions with high values of MHW continue to be predominantly located in Turpan and the Kumtag Desert. As heatwave severity increase, the number of heatwave days decreases, and the affected area shrinks further. The affect area of SHW is reduced, particularly in the Junggar Basin (Figs. 2c). The number of SHW days is distributed between 0 and 14 days, and the area with more than 12 days is only distributed in Turpan. In summary, the heatwaves in Xinjiang during the reference period are dominated by LHW and MHW, and the high value regions of different grades of heatwaves are located in Turpan, Hami, and the Kumtag Desert.

The combination of different grades of heatwave days and population produces the PEH for the reference period (Fig. 2d–f). The results show that the population exposure to light, moderate, and severe heatwave (LPEH, MPEH, and SPEH) amount to 85.1 million, 42.1 million, and 20.5 million person-days, respectively. Notably, the spatial pattern of heatwave and PEH are significantly differs due to the effect of the spatial distribution of population. High PEH values are not in regions with frequent heatwaves, but mainly in densely populated regions such as Urumqi, Kashgar and Hotan.

### Model performance evaluation

**Figure 3:**
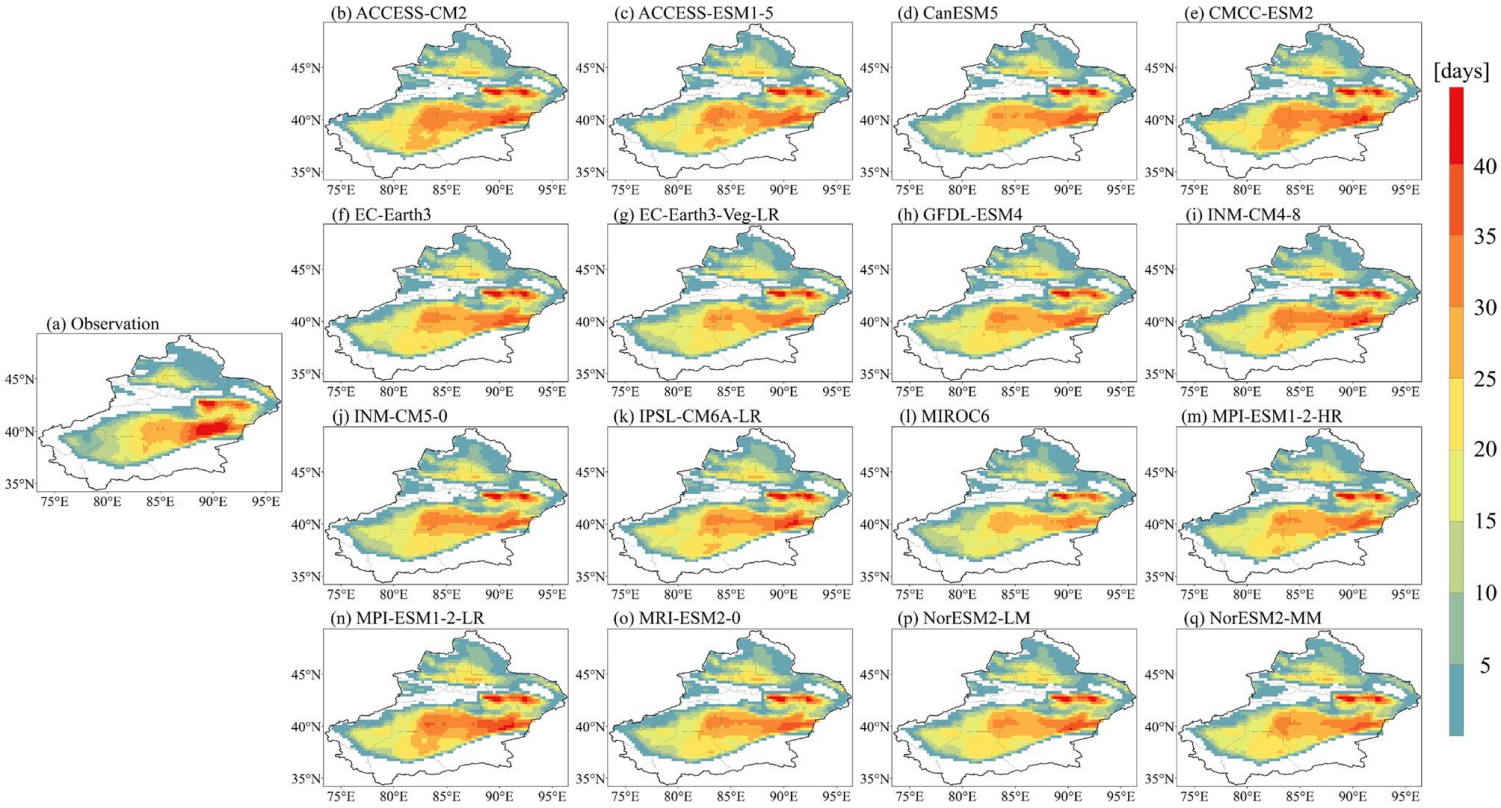
Spatial distribution of (**a**) observed heatwaves and (**b**–**q**) simulated heatwaves for the period 1995–2014.

Before projecting the possible future changes of heatwaves in Xinjiang, the simulation capability of 16 climate models is evaluated by comparing the number of simulated heatwave days with observations during the reference period. As shown in Fig. 3a, the regions in Xinjiang with the highest number of heatwave days are located in the Tuha Basin and Kumutag Desert, and the mountainous regions have never experienced heatwaves. The spatial pattern of the model simulation is close to that of the observations. However, compared with observations, the results of the model simulations are overestimated or underestimated in some regions. To assess the reliability of models, we quantify the ability of individual models to reproduce the interannual variability and spatial patterns of heatwaves in Xinjiang using IVS and DISO, respectively.

**Figure 4:**
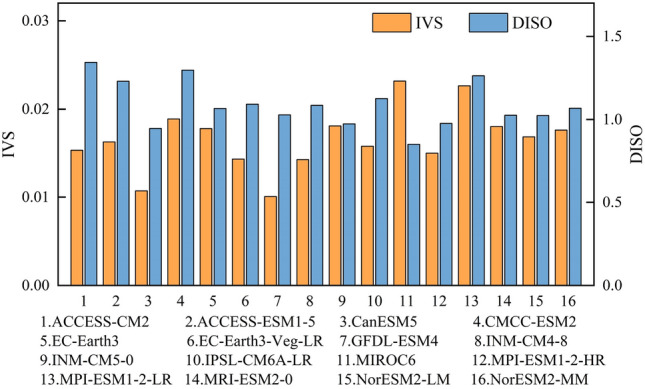
IVS and DISO values for 16 climate models.

As can be seen in Fig. 4, the simulation ability of the models varies in different aspects, with models excelling in simulating interannual variability not necessarily performing better in capturing spatial patterns. Compared to observations, GFDL-ESM4 and MIROC6 are the optimal models for simulating interannual variability and spatial patterns, respectively. In order to assess the comprehensive performance of individual models, a composite ranking of is obtained from their performance in both interannual variability and spatial patterns. Smaller ranking values indicate better model performance. As shown in Table 3, there is substantial variation in the comprehensive performance of models. The highest ranked model is CanESM5, which accordingly is given the highest weight of 0.209 in the combining models.

**Table 3:** Weights and ranks of 16 climate models.

ID	Model name	Rank of IVS	Rank of DISO	Sum of the ranks	Weights
1	ACCESS-CM2	16	6	22	0.038
2	ACCESS-ESM1-5	13	8	21	0.040
3	CanESM5	2	2	4	0.209
4	CMCC-ESM2	15	14	29	0.029
5	EC-Earth3	8	11	19	0.044
6	EC-Earth3-Veg-LR	11	4	15	0.056
7	GFDL-ESM4	7	1	8	0.104
8	INM-CM4-8	10	3	13	0.064
9	INM-CM5-0	3	13	16	0.052
10	IPSL-CM6A-LR	12	7	19	0.044
11	MIROC6	1	16	17	0.049
12	MPI-ESM1-2-HR	4	5	9	0.093
13	MPI-ESM1-2-LR	14	15	29	0.029
14	MRI-ESM2-0	6	12	18	0.046
15	NorESM2-LM	5	9	14	0.060
16	NorESM2-MM	9	10	19	0.044

**Figure 5:**
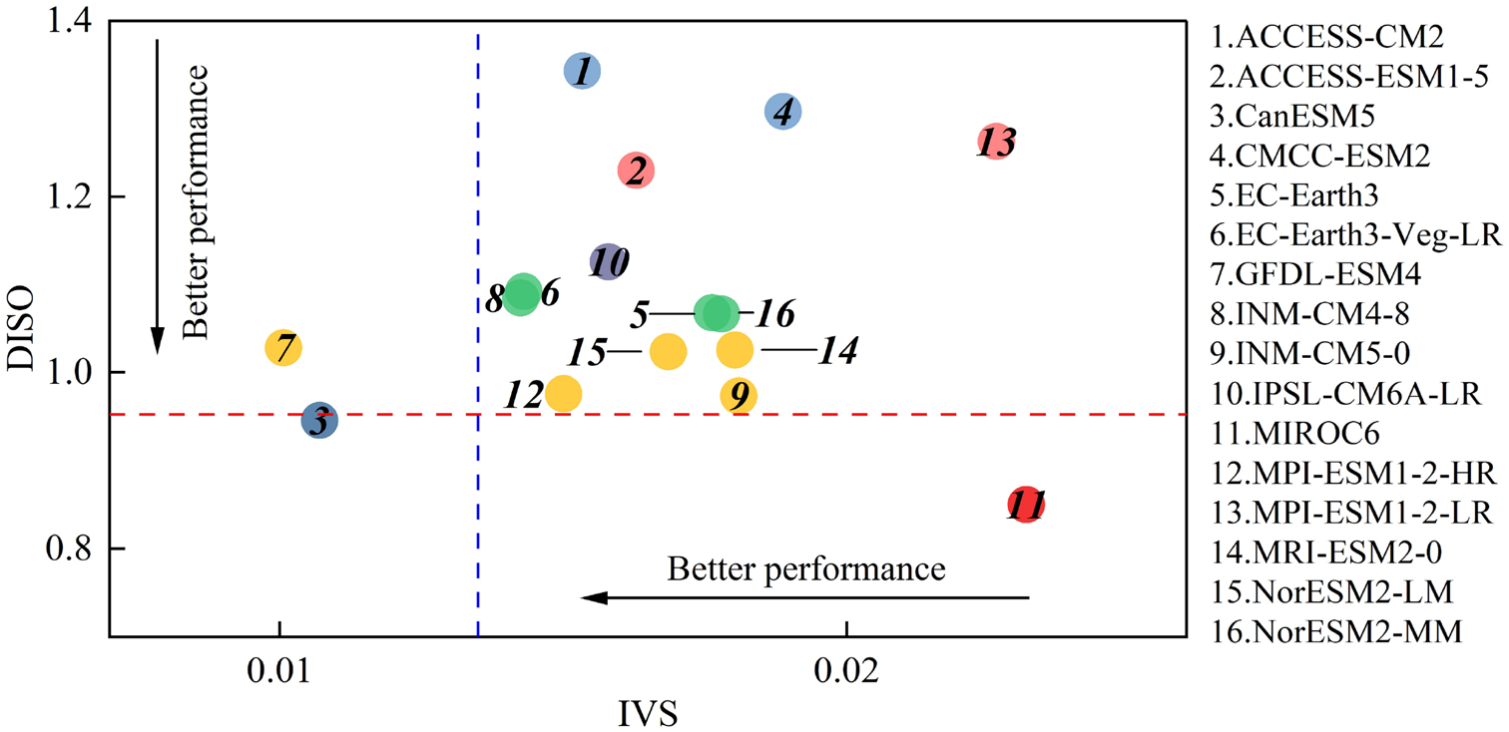
Scatter diagram of the model performance.

Figure 5 compares the performance of MME and each model within the ensemble in simulating the spatial patterns and interannual variability of heatwaves. The blue dashed line and the red dotted line represent the IVS and DISO values of the MME, respectively. Models positioned to the left of the blue dashed line exhibit better performance than the MME in reproducing interannual variability, while models below the red dashed line demonstrate superior performance in capturing spatial patterns. The results show that, among the 16 models, only CanESM5 and GFDL-ESM4 outperform MME in simulating the interannual variability of heatwaves. The MME outperforms the rest of the models, except CanESM5 and MIROC6, in reproducing spatial patterns. Therefore, the MME excels over most models and will be used in this study to represent changes in heatwave projections.

### Projected changes in heatwave

**Figure 6:**
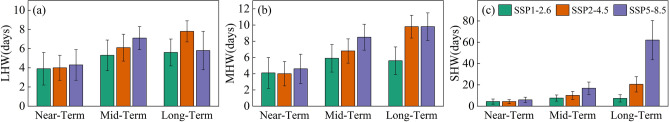
Projected changes in the (**a**) light heatwaves, (**b**) moderate heatwaves and (**c**) severe heatwaves from the MME under different SSP scenarios, relative to the reference period (1995–2014). The colored bars are based on MME, and error bars indicate the standard deviations of the multi-model ensemble projections.

In comparison to the reference period, the number of different grades of heatwave days is anticipated to increase in the future under three SSP scenarios (Fig. 6). The count of LHW days continues to increase under the SSP1-2.6 and SSP2-4.5 scenarios, with the substantial increase of 7.8 ± 1.1 days (MME ± one standard deviation) in the long-term under the SSP2-4.5 scenario (Fig. 6a). Both MHW (Fig. 6b) and SHW (Fig. 6c) days exhibit consistent increase under each scenario. The largest increase is in the long-term under the SSP5-8.5 scenario, with an increase of 9.8 ± 1.7 days and 62 ± 18.4 days, respectively. It is worth noting that in the long-term under the SSP5-8.5 scenario, heatwaves in Xinjiang may no longer be dominated by LHW and MHW, but by SHW. This suggests that Xinjiang is projected to experience more frequent and intense heatwaves.

**Figure 7:**
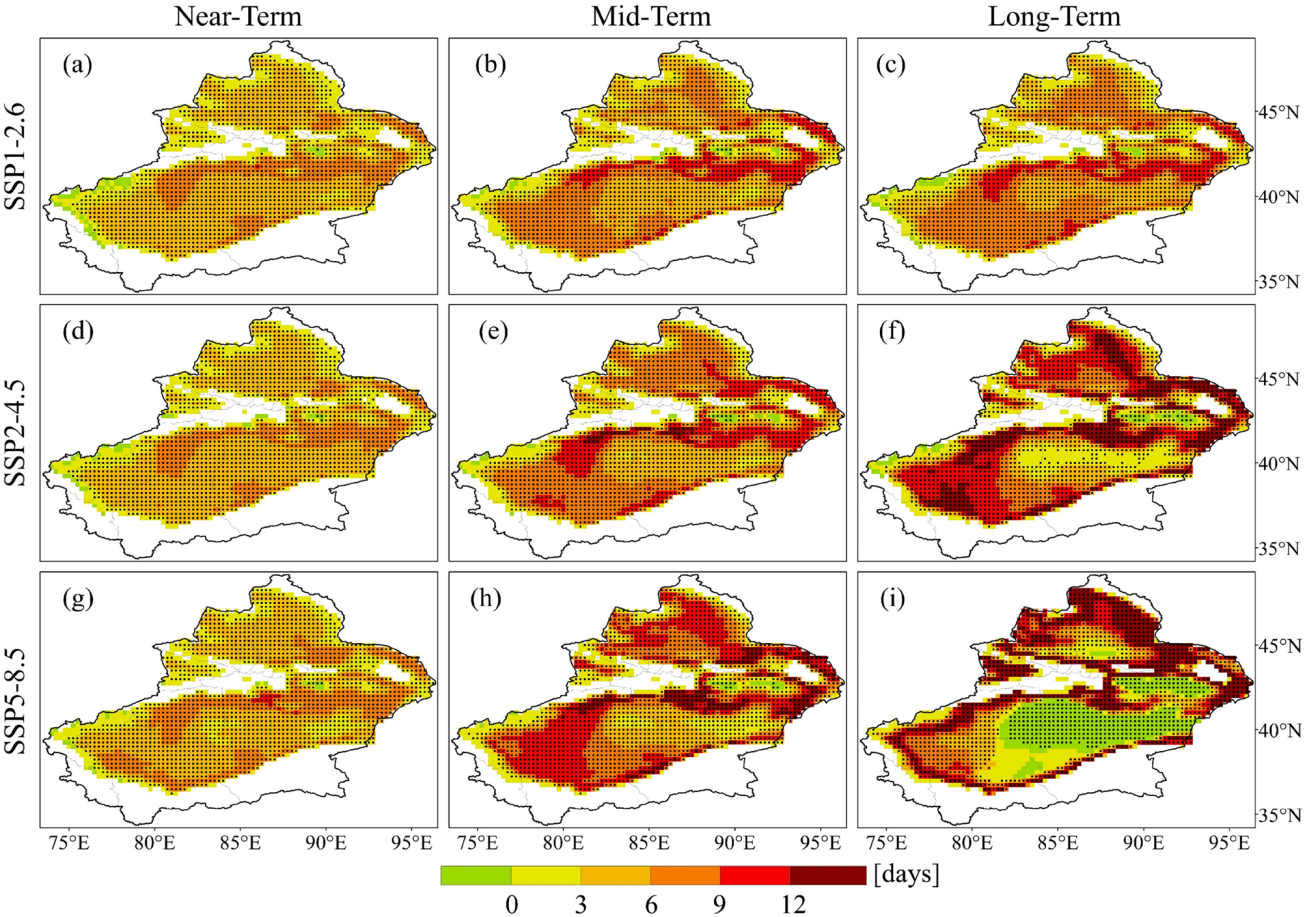
Spatial distribution of projected relative changes of light heatwaves days for different SSP scenarios compared to the reference period. The dotted areas denote regions where at least 75% of models agree with MME on the sign of the change.

The spatial distribution of the variations in heatwave days is crucial for gaining the deeper understanding of the future changes of heatwaves in Xinjiang. Here, the model agreement is denoted by the number of models that have the same sign for changes in heatwave days with the MME results. Compared to the reference period, the number of LHW days increased in most regions of Xinjiang (Fig. 7). Under future scenarios, the greater increases in the number of LHW days are mainly located at the margins of the Tarim Basin and in the northern part of the Junggar Basin, where fewer LHW days occurred during the reference period. Notably, in the long-term under the SSP5-8.5 scenario, there is a substantial decrease in the number of LHW days, predominantly located in the Tuha Basin and the Tarim Basin. In general, areas with large variations in the number of LHW days exhibit higher model agreement.

The number of MHW days is projected to increase in most of Xinjiang under future scenarios compared to the reference period, except for decrease in MHW days in parts of the Tuha Basin in the long-term under the SSP5-8.5 scenario (Fig. S1). Different from LHW and MHW, the number of days of SHW will increase in almost all regions (Fig. S2). Not only is the increase in the number of SHW days significant, but the regions experiencing the greatest increase are primarily located in the regions with the highest number of heatwaves during the reference period. Regions with large increases in the number of MHW and SHW days have high model agreement. In summary, in comparison to the reference period, the area of heatwaves in Xinjiang is expanding, the number of heatwave days is increasing, and the severity of heatwaves is intensifying under different SSP scenarios.

### Estimation of population exposure to future heatwaves

**Figure 8:**
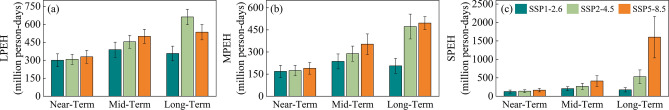
Population exposure of (**a**) light heatwaves, (**b**) moderate heatwaves and (**c**) severe heatwaves under different SSP scenarios. The colored bars are based on MME, and error bars indicate the standard deviations of the multi-model ensemble projections.

To evaluate the population exposed to heatwaves under different SSP scenarios, we calculate PEH for different grades by combining the number of heatwave days with the projected population. Illustrated in Fig. 8, under the SSP1-2.6 scenario, the maximum of LPEH (Fig. 8a), MPEH (Fig. 8b), and SPEH (Fig. 8c) occurs in the mid-term, reaching 388.5 ± 62.9 million (MME ± one standard deviation), 236.1 ± 50.9 million, and 205.2 ± 57.1 million person-days, respectively. Under the SSP2-4.5 and SSP5-8.5 scenarios, the different grades of PEH continued to increase over time. Furthermore, SPEH exceeded the sum of LPEH and MPEH in the long-term under the SSP5-8.5 scenario, at 1602.4 ± 562.5 million person-days.

**Figure 9:**
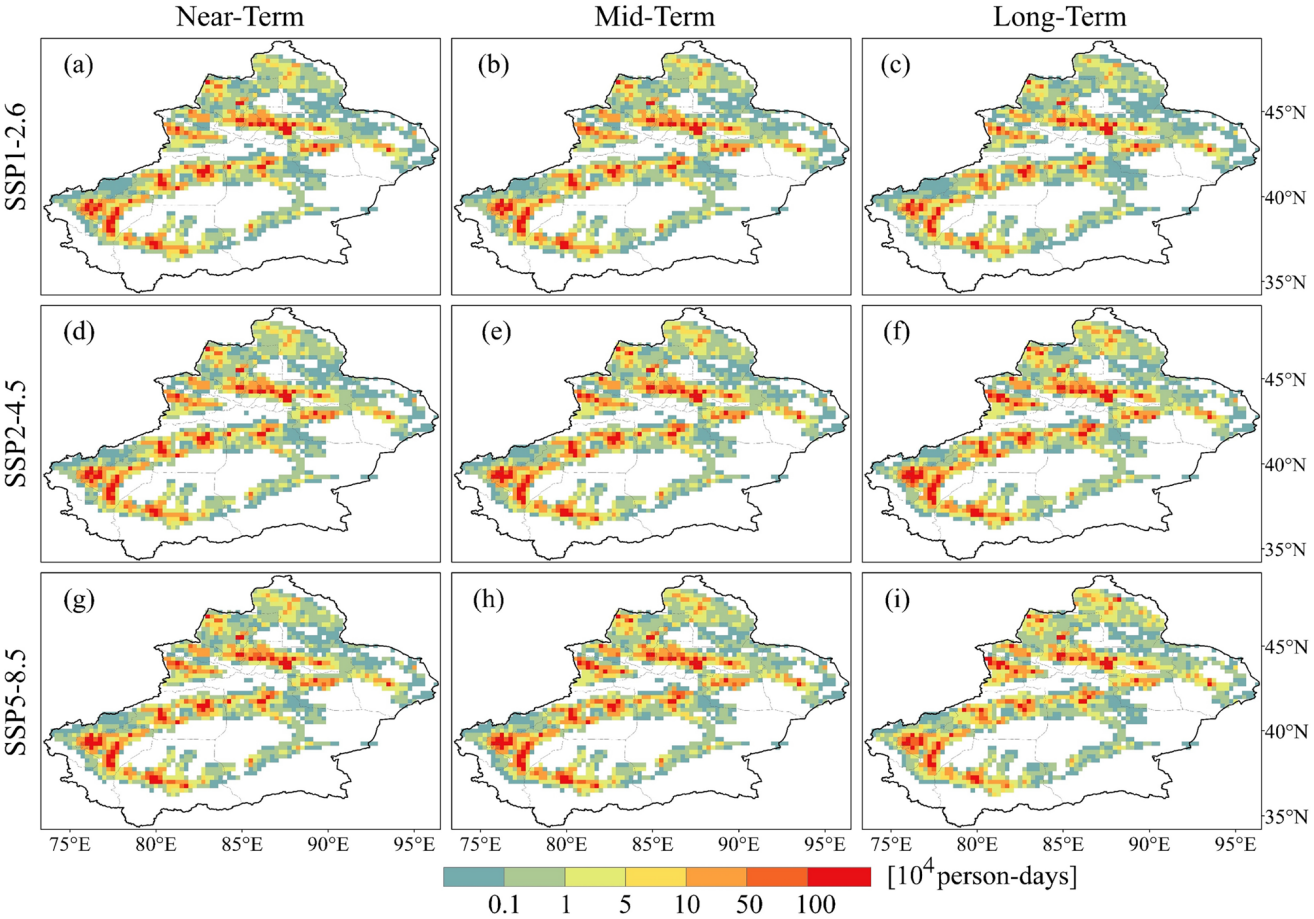
Spatial distribution of population exposure to light heatwaves in future periods under different SSP scenarios.

**Figure 10:**
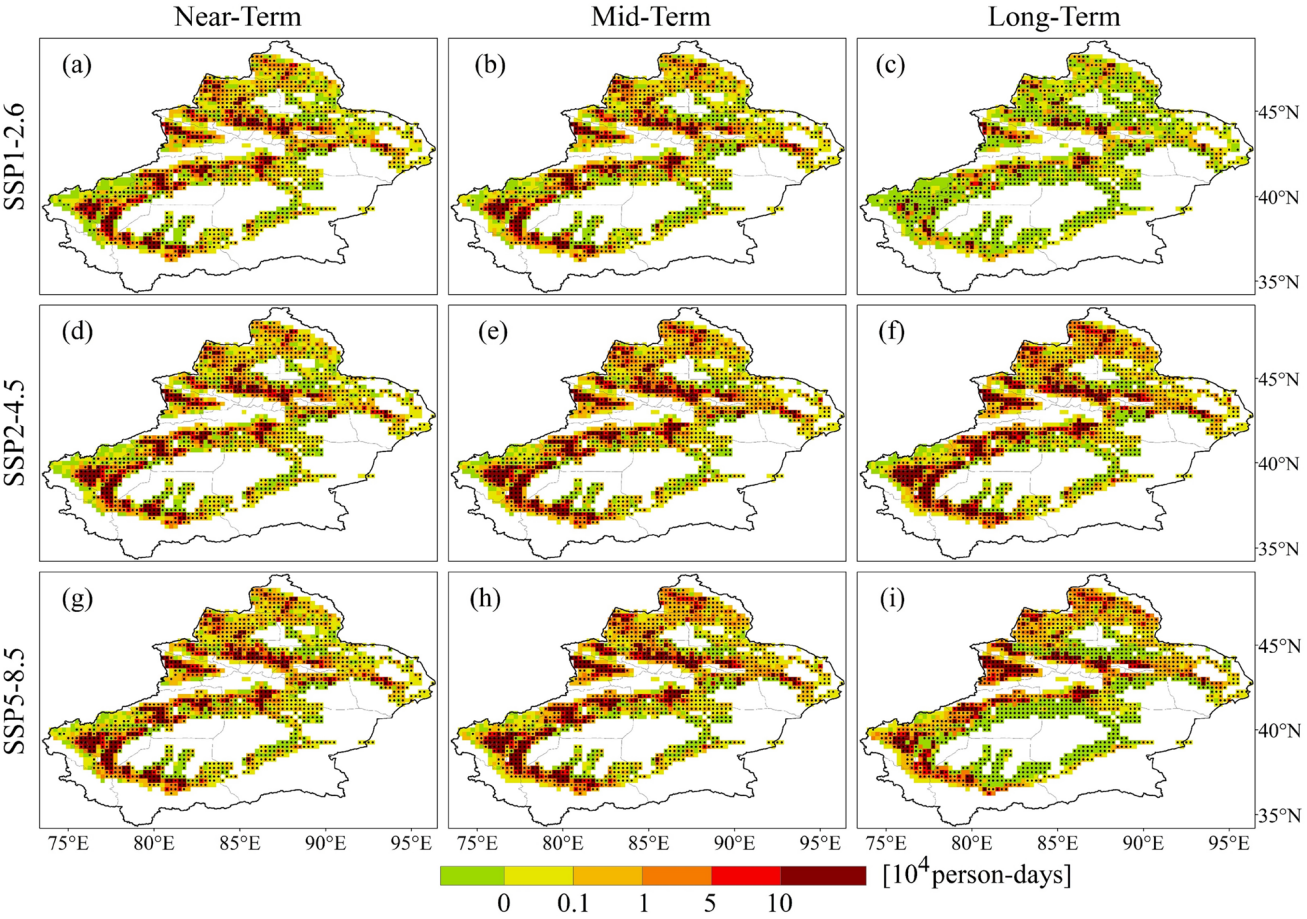
Spatial distribution of projected relative changes in population exposure to light heatwaves for different SSP scenarios compared to the reference period. The dotted areas denote regions where at least 75% of models agree with MME on the sign of the change.

**Figure 11:**
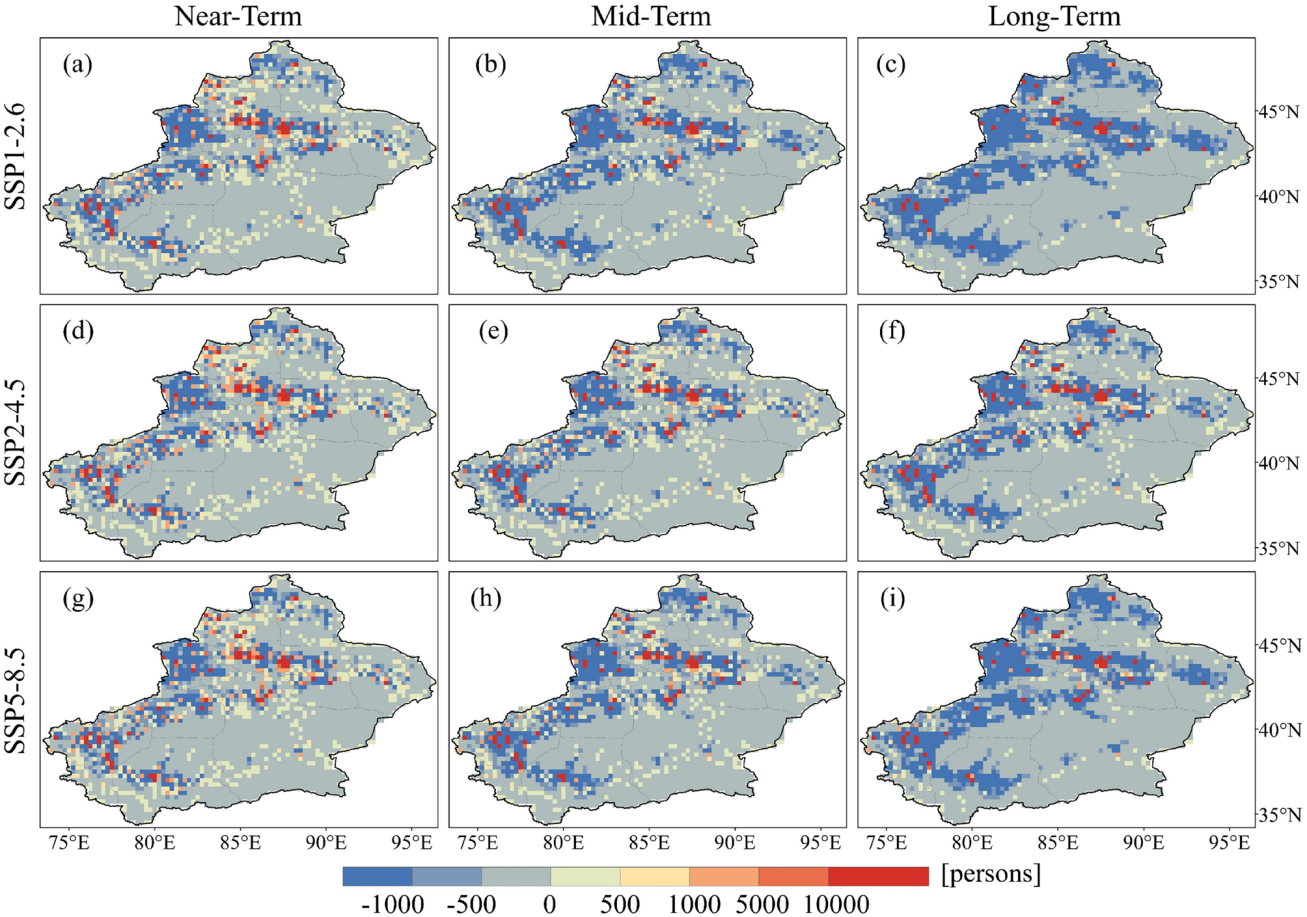
Spatial distribution of projected relative population changes for different SSP scenarios compared to the reference period.

The spatial patterns of the projected PEH resemble those of the reference period, indicating significant spatial divergence. Regions with high value of LPEH during the future period align with those in the reference period, primarily located in Urumqi, Kashgar and Hotan (Fig. 9). In addition, regions that experienced no LPEH during the reference period are projected to exhibit LPEH in the future, such as Kizilsu and the southern parts of Yili. It is important to note that while the total LPEH in the projection period is greater than that during the reference period, not all regions. LPEH decreases in most regions of Xinjiang, and the regions that increase are mainly located in regions with larger LPEH during the reference period, with high model agreement for this change (Fig. 10). The regions with LPEH decreases are more extensive in the long-term under the SSP1-2.6 and SSP5-8.5 scenarios. Correspondingly, the population in most regions of Xinjiang reduces in comparison to the reference period in the long-term under the SSP1-2.6 and SSP5-8.5 scenarios (Fig. 11).

The spatial patterns of projected MPEH (Fig. S3) and SPEH (Fig. S4) are similar to those of the projected LPEH, with nearly identical distributions in both high and low value regions. Compared to the reference period, not only the range of MPEH (Fig. S5) and SPEH (Fig. S6) will expand, but exposure will increase in most regions. In summary, it is projected that more people will be affected by the serious heatwave. The increase in PEH will pose serious threat to future ecosystems and social development. In order to mitigate this threat, it is crucial to understand the effects of changes in PEH.

### Relative contributions of climate and population changes

**Figure 12:**
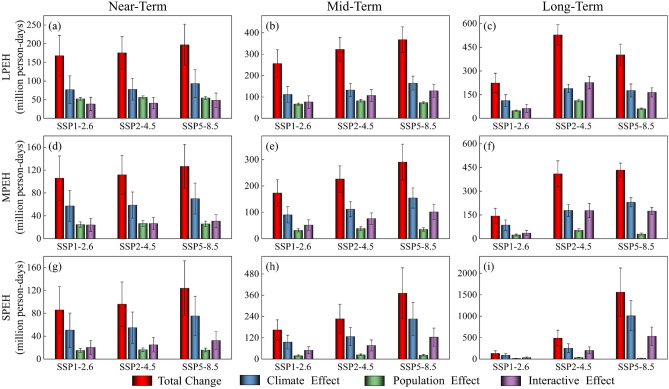
Decomposition of the changes in population exposure to heatwaves in future period under different SSP scenarios. The colored bars are based on MME, and error bars indicate the standard deviations of the multi-model ensemble projections.

Changes in PEH are influenced by climate, population and their interactions. To investigate the relative importance of each factor, we assessed the change in PEH and the relative contribution of the factors for each future period compared to the reference period under different SSP scenarios (Fig. 12 and Table 4). Compared to the reference period, the largest increase in LPEH occurred in the long-term under the SSP2-4.5 scenario, amounting to 528.8 ± 64.4 million person-days. The factor contributing significantly to the increase is the interactive effect, with a contribution of approximately 42.8%, followed by the climate effect, with approximately 35.8%, and lastly, the population effect, with approximately 21.4%. MPEH and SPEH increase the largest in the long-term under the SSP5-8.5 scenario, amounting to 433.5 ± 44.6 million and 1561.1 ± 562.5 million person-days, respectively. The major factor contributing to this change is the climate effect, with contribution of about 53.1% and 64.7%, respectively. In summary, the primary driver of PEH changes in Xinjiang are climate effects, followed by interactive effects, with population effects contributing the least.

**Table 4:** Relative contribution of population, climate, and their interactions to changes in exposure under different SSP scenarios.

Population exposure to heatwaves	SSP-RCP	Future period	Total increase relative to reference period (million person-days)	Increase due to climate effect (%)	Increase due to population effect (%)	Increase due to interactive effect (%)
LPEH	SSP1-2.6	Near-term	167.8	45.9	31.2	22.9
		Mid-term	256	43.9	26.2	29.9
		Long-term	224.3	50.2	21.7	28.1
	SSP2-4.5	Near-term	175.4	44.5	32.2	23.3
		Mid-term	322.3	41.2	25.5	33.3
		Long-term	528.8	35.8	21.4	42.8
	SSP5-8.5	Near-term	196.9	47.4	27.8	24.8
		Mid-term	367.6	44.9	20.1	35.0
		Long-term	402.1	43.9	15.2	40.9
MPEH	SSP1-2.6	Near-term	105.9	54.1	23.3	22.6
		Mid-term	173.5	52.2	18.2	29.6
		Long-term	143.4	59.6	16.0	24.4
	SSP2-4.5	Near-term	111.8	52.6	23.8	23.6
		Mid-term	226.1	49.3	17.2	33.5
		Long-term	409.9	43.6	13.0	43.4
	SSP5-8.5	Near-term	126.7	55.4	20.4	24.2
		Mid-term	290.5	53.1	12.0	34.9
		Long-term	433.5	53.1	6.7	40.2
SPEH	SSP1-2.6	Near-term	85.8	59.0	17.4	23.6
		Mid-term	164	58.2	11.6	30.2
		Long-term	133	65.9	9.8	24.3
	SSP2-4.5	Near-term	96	57.1	16.9	26.0
		Mid-term	226.6	55.8	10.5	33.7
		Long-term	488	51.9	6.7	41.4
	SSP5-8.5	Near-term	123.9	61.0	12.8	26.2
		Mid-term	371	61.0	5.7	33.3
		Long-term	1561.1	64.7	1.1	34.2

## Conclusion and discussion

Xinjiang is an important part of the global arid zone, experiences frequent heatwaves. The frequency of heatwaves in Xinjiang has significantly increased with climate change, posing the serious threat to human health^51^. Therefore, projecting the impact of heatwaves on human health in Xinjiang is the crucial and pressing task. In considering the effects of heatwaves on human health, it is essential to not only focus on the heatwaves but also population dynamic. Currently, most population data used in projecting future population exposure studies do not account for changes in China's population policy, potentially leading to biased projections^52^. To enhance the accuracy of PEH projections, we utilize the dataset that incorporates recent changes in China's population-related policies, including population ceiling restrictions in megacities and fertility promotion, to project PEH in Xinjiang. Therefore, this study aims to provide the first comprehensive evaluation of the variation in PEH in Xinjiang under climate change by integrating heatwaves and population changes, and to quantitatively assess the contribution of each factor to changes in PEH. The results of this study provide a scientific basis for mitigating heatwave hazards and formulating sustainable development policies.

The spatial distribution of heatwaves in Xinjiang is closely related to topographic features and is predominantly distributed in basin areas, such as the Junggar Basin, the Tarim Basin and the Tuha Basin. This is primarily due to the geographical characteristics of the basins, which contribute to the occurrence and intensification of heatwaves. For instance, the lower elevation, higher temperatures and humidity of these basins render those regions susceptible to heatwaves. The topography of the basins restricts air circulation, leading to the trapping of heat and further intensification of heatwaves. Additionally, the predominantly desert and semi-desert land cover types in these areas make them more prone to heatwaves. During the reference period, heatwaves in Xinjiang are dominated by LHW and MHW. This is consistent with the results obtained by Liu et al.^53^ who examined various grades of heatwaves in China. In terms of area affected, LHW is the largest, followed by MHW, and SHW is the smallest, with the most significantly change in Yili.

The spatial distribution of PEH is significantly different from that of heatwaves. Regions with higher number of heatwave days do not necessarily have higher PEH or may not have it, such as the Kumtag Desert. The spatial distribution of PEH is determined by both heatwaves and population, and the geographical distribution pattern of the Mountain-Desert-Oasis system determines that the population of Xinjiang is mainly concentrated in oases^54^. Therefore, regions with higher PEH values are mainly located in densely populated areas such as Urumqi, Kashgar and Hotan. Since most areas in Xinjiang are susceptible to heatwaves, the spatial distribution of PEH depends mainly on that of population.

By evaluating the simulation ability of individual models, it is found that GFDL-ESM4 and MIROC6 are optimal models for capturing interannual variability and spatial patterns of heatwaves in Xinjiang, respectively. However, the highest overall ranking among the ensemble models is CanESM5. Weighting individual model members within the ensemble based on their performance is considered as a way to reduce uncertainty^55^. Considering the differences in model performance, and in order to provide more reasonable projections, we evaluate the simulation capabilities of the MME. The comparison reveals that the overall simulation capability of MME outperforms all the remaining models in the ensemble except CanESM5. Individual model projections are affected by higher internal climate variability making the projections more uncertain than MME^56^. Therefore, in order to provide more reasonable information and uncertainty in the projection, this study relies on MME to project future heatwaves and PEH variations in Xinjiang.

Compared with the reference period, the total number of heatwave days in Xinjiang is projected to increase, aligning with findings from previous studies^37,57^. However, using only air temperature to identify heatwaves in their study ignored the effect of relative humidity on human health. The combination of high temperatures and high relative humidity can influence the heat dissipation capabilities of the human body. Relying on temperature may lead to an underestimation of the impact of the environment on human health^58^. Although the majority of heatwaves in Xinjiang are categorized as dry heatwaves, the consideration of combined air temperature and relative humidity are necessary under the climate become wetter^59,60^. In addition, different from the finding that the increase in heatwaves grows sequentially from SSP1-2.6 to SSP5-8.5, the increase in LHW and MHW in the long-term under the SSP5-8.5 scenario is smaller than that of under the SSP2-4.5 scenario^61^. This discrepancy is primarily attributed to the substantial increase in SHW. Consequently, the heatwaves in Xinjiang are projected to be more serious in the long-term under the SSP5-8.5 scenario.

Spatially, the region affected by heatwaves in Xinjiang is projected to expand under different SSP scenarios. Despite the overall expansion of heatwaves, the mountains regions remain unaffected by heatwave in all scenarios. This inconsistent with previous studies projecting heatwaves in China that found heatwaves in the mountains of Xinjiang in the future^36^. The discrepancy arises from the chosen method for identifying heatwaves. Our study calculated the HI for samples with daily maximum temperatures exceeding 33 ℃, effectively excluding colder regions where heatwaves are less likely to occur. Consistent with previous studies projecting an increase in heatwave severity across most regions of Xinjiang^57^, our findings project that most regions of Xinjiang will experience frequent SHW in the long-term under the SSP5-8.5 scenario. Furthermore, regions with decrease in the number of LHW days are characterized by an increase in MHW and SHW.

Climate change and population growth are projected to result in an increase in PEH in Xinjiang. The maximum values of PEH occur in the mid-term under the SSP1-2.6 scenario. This may be due to the SSP1-2.6 scenario represents a sustainable development scenario, which is characterized by slower population growth and lower greenhouse gas emissions^62^. Different grades of PEH continue to increase over time and reach the maximum in the long-term under the SSP2-4.5 and SSP5-8.5 scenarios. Under the SSP2-4.5 scenario, future fertility rate is moderate due to the effect of two-child policy, while the SSP5-8.5 scenario exhibits lower fertility^46^. Thus, the contribution of population effects to changes in PEH is consistently higher under the SSP2-4.5 scenario than SSP5-8.5. Compared with SSP5-8.5, Xinjiang has more population but less PEH under the SSP2-4.5 scenario. The results suggest that changes in PEH in Xinjiang are more sensitive to climate change than to variations in population.

In terms of spatial distribution, the spatial pattern of PEH during the projection period mirrors that observed in the reference period. High PEH values are still in the densely populated areas such as Hotan, Kashgar and Urumqi. Although the total PEH during the projection period exceeds that of the reference period, spatially, many regions show a decrease in PEH compared to the reference period. Under the SSP1-2.6 scenario, PEH decreases in many regions, but the number of heatwave days does not reduce in these regions. Thus, the decrease in PEH under the SSP1-2.6 scenario is mainly the result of lower fertility and mortality rates^63^. However, the reduction in population is not only related to fertility and mortality rates, but also to migration. Under the SSP5-8.5 scenario, with social and economic development, population concentrates in cities. Therefore, under the SSP5-8.5 scenario, the total number is increasing, although exposure is projected to decrease in many regions. Changes in PEH are influenced by climate, population and their interactions. Understanding the relative importance of factors influencing changes in PEH is crucial for developing climate change adaptation and mitigation policies in the study area. Since the primary driver of PEH change in Xinjiang is climate effect. Thus, consistent with the findings of Li et al.^64^, climate mitigation is particularly important in order to reduce population exposure to unprecedented heatwaves.

### Supplementary Information

Supplementary Figures.

## Data Availability

The data that support the findings of this study are available from the corresponding author upon reasonable request.

## References

[1] Perkins-Kirkpatrick SE, Lewis SC (2020). Increasing trends in regional heatwaves. Nat. Commun..

[2] Al-Yaari A, Zhao Y, Cheruy F, Thiery W (2023). Heatwave characteristics in the recent climate and at different global warming levels: A multimodel analysis at the global scale. Earth's Future.

[3] Robine J-M, Cheung SLK, Le Roy S, Van Oyen H, Griffiths C, Michel J-P, Herrmann FR (2008). Death toll exceeded 70,000 in Europe during the summer of 2003. Comptes Rend. Biol..

[4] Yan M, Xie Y, Zhu H, Ban J, Gong J, Li T (2022). The exceptional heatwaves of 2017 and all-cause mortality: An assessment of nationwide health and economic impacts in China. Sci. Total Environ..

[5] Tuholske C, Caylor K, Funk C, Verdin A, Sweeney S, Grace K, Peterson P, Evans T (2021). Global urban population exposure to extreme heat. Proc. Natl. Acad. Sci..

[6] Cardona OD (2012). Managing the Risks of Extreme Events and Disasters to Advance Climate Change Adaptation.

[7] Jones B, Tebaldi C, O’Neill BC, Oleson K, Gao J (2018). Avoiding population exposure to heat-related extremes: Demographic change vs climate change. Clim. Change.

[8] Wen S, Wang A, Tao H, Malik K, Huang J, Zhai J, Jing C, Rasul G, Su B (2019). Population exposed to drought under the 1.5 °C and 2.0 °C warming in the Indus River Basin. Atmos. Res..

[9] Tellman B, Sullivan JA, Kuhn C, Kettner AJ, Doyle CS, Brakenridge GR, Erickson TA, Slayback DA (2021). Satellite imaging reveals increased proportion of population exposed to floods. Nature.

[10] Shen L, Wen J, Zhang Y, Ullah S, Cheng J, Meng X (2022). Changes in population exposure to extreme precipitation in the Yangtze River Delta, China. Clim. Serv..

[11] Chambers J (2020). Global and cross-country analysis of exposure of vulnerable populations to heatwaves from 1980 to 2018. Clim. Change.

[12] Chen H, Sun J (2021). Significant increase of the global population exposure to increased precipitation extremes in the future. Earth's Future.

[13] Wang Y, Zhao N, Yin X, Wu C, Chen M, Jiao Y, Yue T (2023). Global future population exposure to heatwaves. Environ. Int..

[14] Jones B, O’Neill BC, McDaniel L, McGinnis S, Mearns LO, Tebaldi C (2015). Future population exposure to US heat extremes. Nat. Clim. Change.

[15] Sun H, Wang Y, Chen J, Zhai J, Jing C, Zeng X, Ju H, Zhao N, Zhan M, Luo L (2017). Exposure of population to droughts in the Haihe River Basin under global warming of 1.5 and 2.0 °C scenarios. Quat. Int..

[16] Lyon B, Barnston AG, Coffel E, Horton RM (2019). Projected increase in the spatial extent of contiguous US summer heat waves and associated attributes. Environ. Res. Lett..

[17] Liu Y, Chen J, Pan T, Liu Y, Zhang Y, Ge Q, Ciais P, Penuelas J (2020). Global socioeconomic risk of precipitation extremes under climate change. Earths Future.

[18] Mora C, Dousset B, Caldwell IR, Powell FE, Geronimo RC, Bielecki CR, Counsell CWW, Dietrich BS, Johnston ET, Louis LV (2017). Global risk of deadly heat. Nat. Clim. Change.

[19] Coffel ED, Horton RM, de Sherbinin A (2018). Temperature and humidity based projections of a rapid rise in global heat stress exposure during the 21(st) century. Environ. Res. Lett..

[20] King AD, Harrington LJ (2018). The inequality of climate change from 1.5 to 2°C of global warming. Geophys. Res. Lett..

[21] Russo S, Sillmann J, Sippel S, Barcikowska MJ, Ghisetti C, Smid M, O'Neill B (2019). Half a degree and rapid socioeconomic development matter for heatwave risk. Nat. Commun..

[22] Ding T, Qian W, Yan Z (2010). Changes in hot days and heat waves in China during 1961–2007. Int. J. Climatol..

[23] Liu X, Tang Q, Zhang X, Sun S (2018). Projected changes in extreme high temperature and heat stress in China. J. Meteorol. Res..

[24] Sun J (2014). Record-breaking SST over mid-North Atlantic and extreme high temperature over the Jianghuai-Jiangnan region of China in 2013. Chin. Sci. Bull..

[25] Harrington LJ, Otto FEL (2018). Changing population dynamics and uneven temperature emergence combine to exacerbate regional exposure to heat extremes under 1.5 °C and 2 °C of warming. Environ. Res. Lett..

[26] Luo M, Lau NC (2018). Increasing heat stress in urban areas of Eastern China: Acceleration by urbanization. Geophys. Res. Lett..

[27] Luo M, Lau N-C (2017). Heat waves in Southern China: Synoptic behavior, long-term change, and urbanization effects. J. Clim..

[28] Zhang J, You Q, Ren G, Ullah S (2023). Substantial increase in human-perceived heatwaves in eastern China in a warmer future. Atmos. Res..

[29] Liang L, Chen M, Luo X, Xian Y (2021). Changes pattern in the population and economic gravity centers since the Reform and Opening up in China: The widening gaps between the South and North. J. Clean. Prod..

[30] Zhang G-W, Zeng G, Iyakaremye V, You Q-L (2020). Regional changes in extreme heat events in China under stabilized 1.5 °C and 2.0 °C global warming. Adv. Clim. Change Res..

[31] Luo M, Ning G, Xu F, Wang S, Liu Z, Yang Y (2020). Observed heatwave changes in arid northwest China: Physical mechanism and long-term trend. Atmos. Res..

[32] Toops S (2016). Reflections on China's belt and road initiative. Area Dev. Policy.

[33] Yao J, Chen Y, Zhao Y, Mao W, Xu X, Liu Y, Yang Q (2017). Response of vegetation NDVI to climatic extremes in the arid region of Central Asia: A case study in Xinjiang, China. Theor. Appl. Climatol..

[34] Dong D, Tao H, Ding G, Zhang Z (2022). Historical population and cropland exposure to heatwaves in Xinjiang, China. Trans. Chin. Soc. Agric. Eng..

[35] Shang L, Huang Y, Maoweiyi.  (2016). Features of the snow and ice meltwater flood caused by high temperature in the Southern Xinjiang Region during the summer of 2015. J. Glaciol. Geocryol..

[36] Wei J, Wang W, Wang G, Cao M, Yang L, Zhang S, Fu J, Xing W (2023). Projecting the changes in multifaceted characteristics of heatwave events across China. Earth's Future.

[37] Yang Y, Jin C, Ali S (2020). Projection of heat wave in China under global warming targets of 1.5 °C and 2 °C by the ISIMIP models. Atmos. Res..

[38] Shi Y, Shen Y, Kang E, Li D, Ding Y, Zhang G, Hu R (2006). Recent and future climate change in Northwest China. Clim. Change.

[39] Wu Z, Zhang H, Krause CM, Cobb NS (2010). Climate change and human activities: A case study in Xinjiang, China. Clim. Change.

[40] Yang Z, Lei J, Duan Z, Dong J, Su C (2016). Spatial distribution of population in Xinjiang. Geogr. Res..

[41] Wu J, Gao X, Giorgi F, Chen D (2017). Changes of effective temperature and cold/hot days in late decades over China based on a high resolution gridded observation dataset. Int. J. Climatol..

[42] Thrasher B, Wang W, Michaelis A, Melton F, Lee T, Nemani R (2022). NASA global daily downscaled projections, CMIP6. Sci. Data.

[43] Ali J, Syed KH, Gabriel HF, Saeed F, Ahmad B, Bukhari SAA (2018). Centennial heat wave projections over Pakistan using ensemble NEX GDDP data set. Earth Syst. Environ..

[44] Shao D, Li H, Wang J, Hao X, Niu L (2023). Adaptability analysis of snow in the Zhangjiakou competition zone of the Beijing Olympic Winter Games for the next 30 years. J. Hydrol. Reg. Stud..

[45] Shiru MS, Shahid S, Chung E-S, Alias N, Scherer L (2019). A MCDM-based framework for selection of general circulation models and projection of spatio-temporal rainfall changes: A case study of Nigeria. Atmos. Res..

[46] Chen Y, Guo F, Wang J, Cai W, Wang C, Wang K (2020). Provincial and gridded population projection for China under shared socioeconomic pathways from 2010 to 2100. Sci. Data.

[47] Huang Z, Chen H, Tian H (2011). Research on the heat wave index. Meteorol. Mon..

[48] Chen W, Jiang Z, Li L (2011). Probabilistic projections of climate change over China under the SRES A1B scenario using 28 AOGCMs. J. Clim..

[49] Hu Z, Chen X, Zhou Q, Chen D, Li J (2019). DISO: A rethink of Taylor diagram. Int. J. Climatol..

[50] Bağçaci SÇ, Yucel I, Duzenli E, Yilmaz MT (2021). Intercomparison of the expected change in the temperature and the precipitation retrieved from CMIP6 and CMIP5 climate projections: A Mediterranean hot spot case, Turkey. Atmos. Res..

[51] Guan J, Yao J, Li M, Li D, Zheng J (2022). Historical changes and projected trends of extreme climate events in Xinjiang, China. Clim. Dyn..

[52] Liao X, Xu W, Zhang J, Li Y, Tian Y (2019). Global exposure to rainstorms and the contribution rates of climate change and population change. Sci. Total Environ..

[53] Liu J, Ren Y, Tao H, Shalamzari MJ (2021). Spatial and temporal variation characteristics of heatwaves in recent decades over China. Remote Sens..

[54] Lu D, Wang Z, Feng Z, Zeng G, Fang C, Dong X, Liu S, Jia S, Fang Y, Meng G (2016). Academic debates on Hu Huanyong population line. Geogr. Res..

[55] Christensen JH, Kjellström E, Giorgi F, Lenderink G, Rummukainen M (2010). Weight assignment in regional climate models. Clim. Res..

[56] Yang Y, Zhang Y, Gao Z, Pan Z, Zhang X (2023). Historical and projected changes in temperature extremes over inconsistency between China and the multimodel ensembles and individual models from CMIP5 and CMIP6. Earth Space Sci..

[57] Guo X, Huang J, Luo Y, Zhao Z, Xu Y (2016). Projection of heat waves over China for eight different global warming targets using 12 CMIP5 models. Theor. Appl. Climatol..

[58] Basu R, Samet JM (2002). Relation between elevated ambient temperature and mortality: A review of the epidemiologic evidence. Epidemiol. Rev..

[59] Xu F, Chan TO, Luo M (2020). Different changes in dry and humid heat waves over China. Int. J. Climatol..

[60] Yao J, Chen Y, Guan X, Zhao Y, Chen J, Mao W (2022). Recent climate and hydrological changes in a mountain–basin system in Xinjiang, China. Earth-Sci. Rev..

[61] Wang Y, Zhao N, Wu C, Quan J, Chen M (2023). Future population exposure to heatwaves in 83 global megacities. Sci. Total Environ..

[62] O'Neill BC, Tebaldi C, van Vuuren DP, Eyring V, Friedlingstein P, Hurtt G, Knutti R, Kriegler E, Lamarque J-F, Lowe J (2016). The scenario model intercomparison project (ScenarioMIP) for CMIP6. Geosci. Model Dev..

[63] O’Neill BC, Kriegler E, Ebi KL, Kemp-Benedict E, Riahi K, Rothman DS, van Ruijven BJ, van Vuuren DP, Birkmann J, Kok K (2017). The roads ahead: Narratives for shared socioeconomic pathways describing world futures in the 21st century. Glob. Environ. Change.

[64] Li M, Zhou B-B, Gao M, Chen Y, Hao M, Hu G, Li X (2022). Spatiotemporal dynamics of global population and heat exposure (2020–2100): Based on improved SSP-consistent population projections. Environ. Res. Lett..

