# Supplementary material for: Projected population exposure to heatwaves in Xinjiang Uygur autonomous region, China
Source: Sci Rep. 2024 Feb 25;14:4570. doi: 10.1038/s41598-024-54885-1 (PMC10894881; doi:10.1038/s41598-024-54885-1)
Supplement: Supplementary file 1 — Supplementary Figures. [file 41598_2024_54885_MOESM1_ESM.docx]

**Projected Population Exposure to Heatwaves in Xinjiang Uygur Autonomous Region, China**

**Diwen Dong^1,2,4,5^, Hui Tao^2^* and Zengxin Zhang^2,3^**

^1^ College of Ecology and Environment, Xinjiang University, Urumqi 830017, China

^2^ State Key Laboratory of Desert and Oasis Ecology, Xinjiang Institute of Ecology and Geography, Chinese Academy of Sciences, Urumqi 830011, China

^3^ Joint Innovation Center for Modern Forestry Studies, College of Forestry, Nanjing Forestry University, Nanjing 210037, China

^4^ Institute of Statistics and Data Science, Xinjiang University of Finance & Economics, Urumqi, 830012, China

^5^ University of Chinese Academy of Sciences, Beijing 100049, China

Corresponding author: Hui Tao ([taohui@ms.xjb.ac.cn](mailto:taohui@ms.xjb.ac.cn))

Contents of this file

Figure S1 to S6


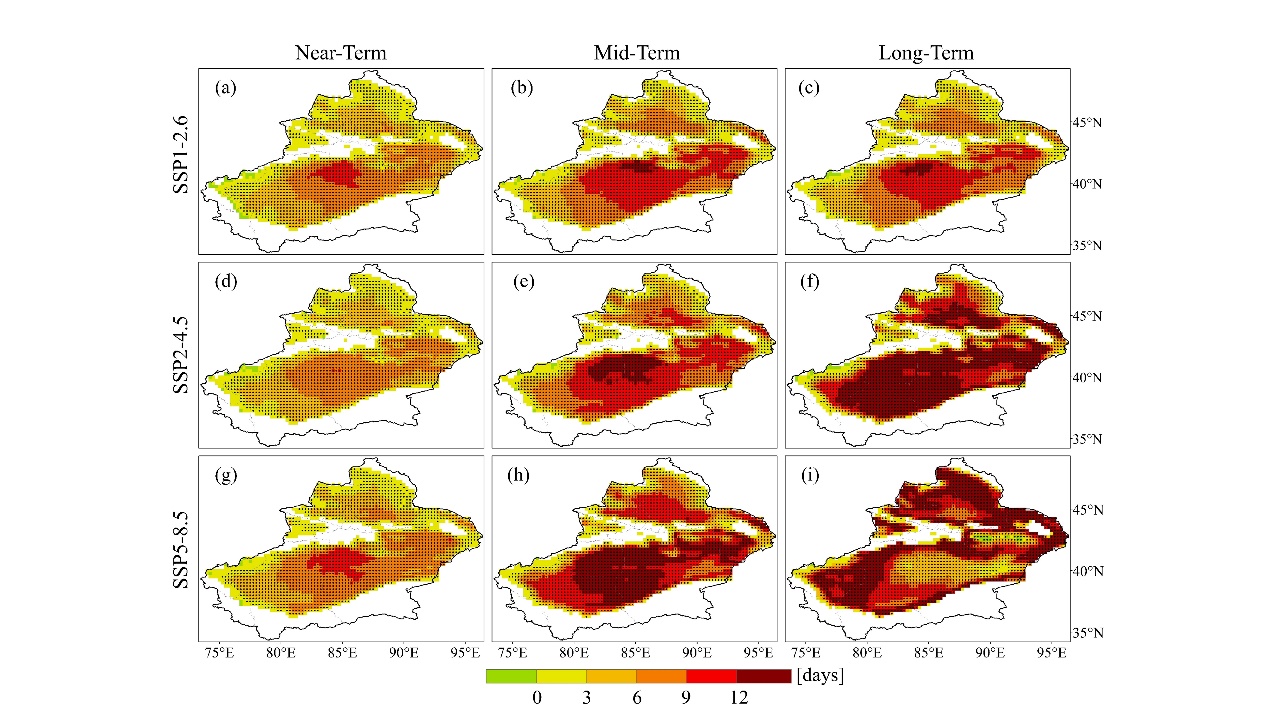


Figure S1. Spatial distribution of projected relative changes of moderate heatwaves days for different SSP scenarios compared to the reference period. The dotted areas denote regions where at least 75% of models agree with MME on the sign of the change.


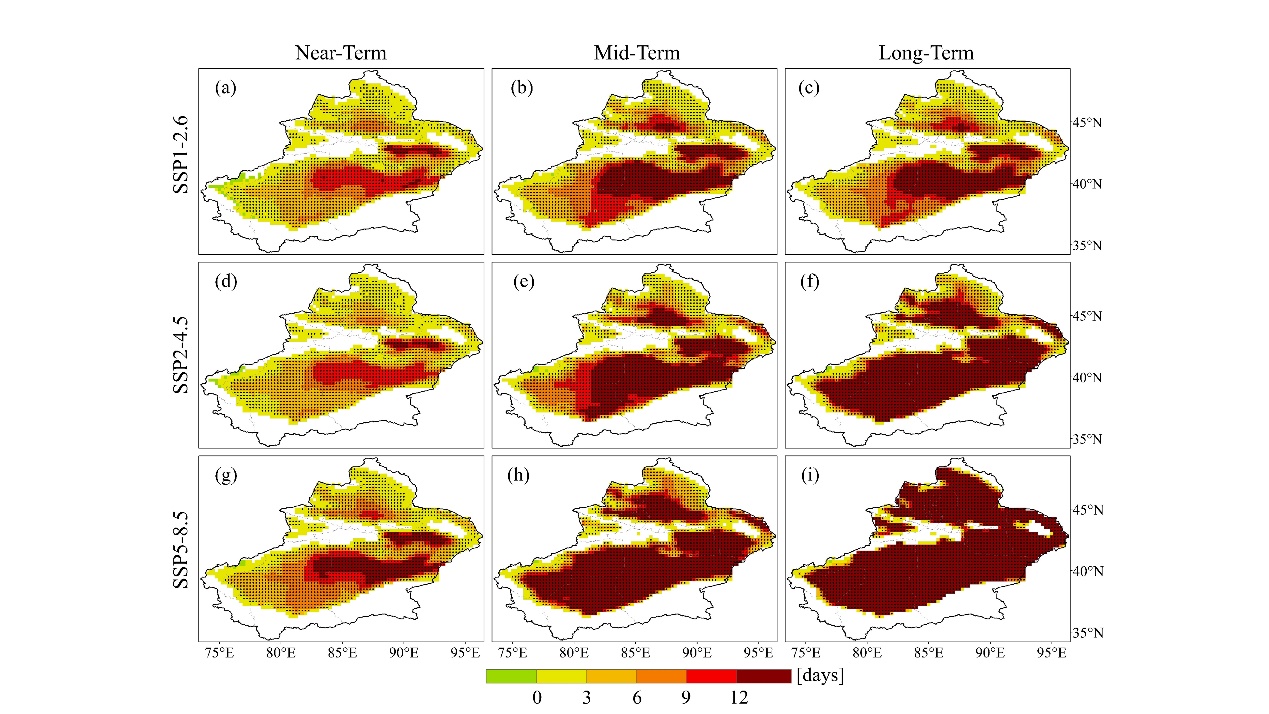


Figure S2. Same as Figure S1, but for severe heatwaves.


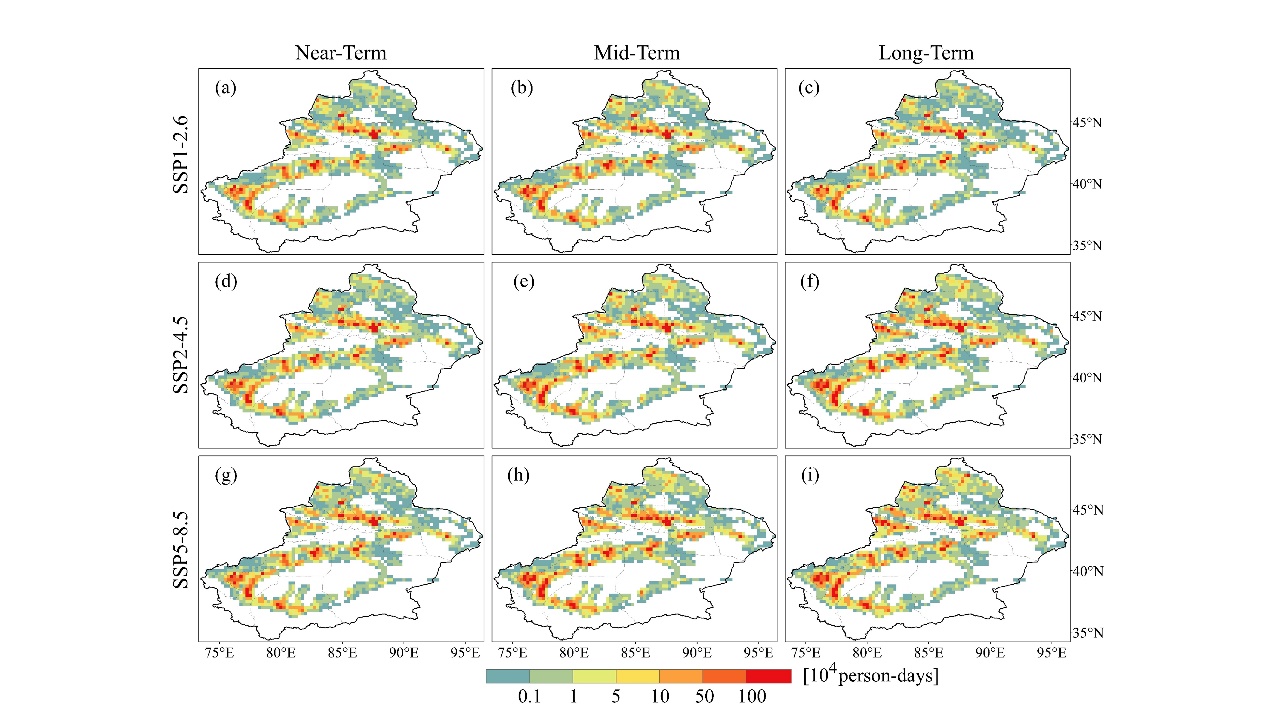


Figure S3. Spatial distribution of population exposure to moderate heatwaves in future periods under different SSP scenarios.


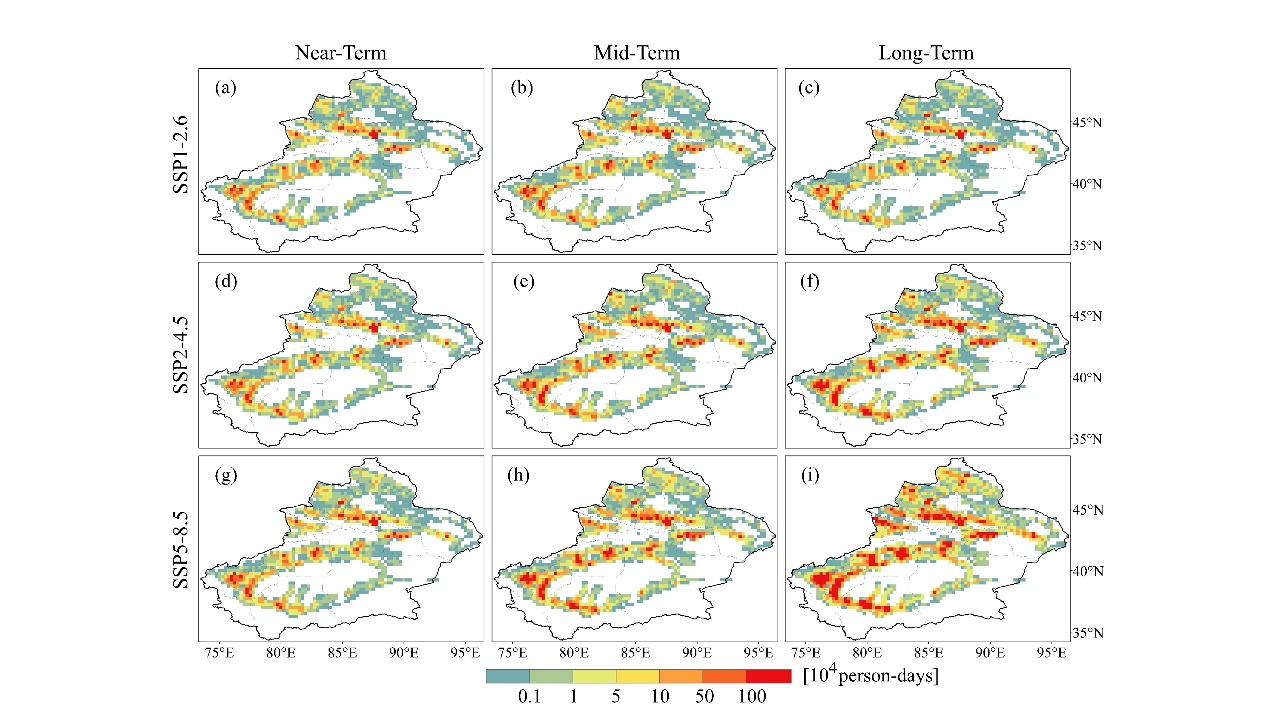


Figure S4. Same as Figure S3, but for severe heatwaves.


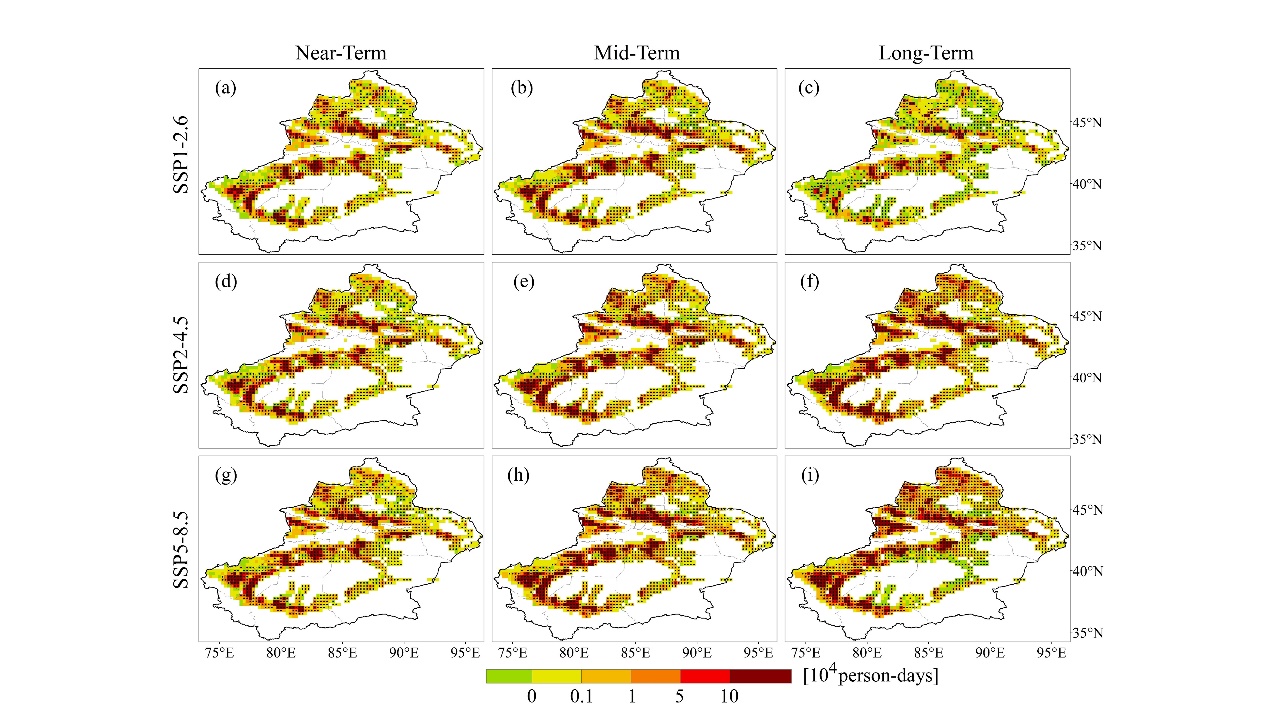


Figure S5. Spatial distribution of projected relative changes in population exposure to moderate heatwaves for different SSP scenarios compared to the reference period. The dotted areas denote regions where at least 75% of models agree with MME on the sign of the change.


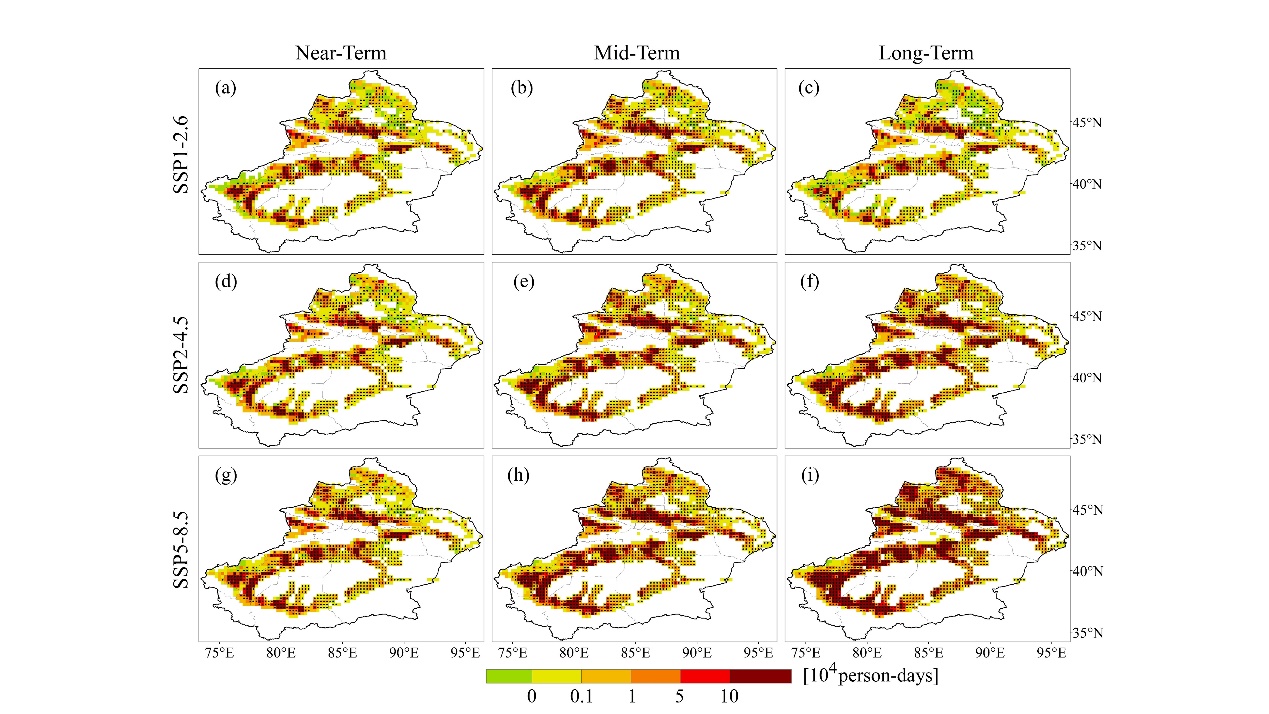


Figure S6. Same as Figure S5, but for severe heatwaves.
